# Physical Activity and the Risk of Hemorrhagic Stroke: A Population-Based Longitudinal Follow-Up Study in Taiwan

**DOI:** 10.3389/fmed.2021.791772

**Published:** 2021-12-23

**Authors:** Shih-Hao Feng, Li-Sheng Chen, Kuo-Cheng Yeh, Shin-Liang Pan

**Affiliations:** ^1^Department of Physical Medicine and Rehabilitation, National Taiwan University BioMedical Park Hospital, Hsinchu, Taiwan; ^2^School of Oral Hygiene, College of Oral Medicine, Taipei Medical University, Taipei, Taiwan; ^3^Departments of Physical Medicine and Rehabilitation, National Taiwan University Hospital, National Taiwan University, Taipei, Taiwan; ^4^College of Medicine, National Taiwan University, Taipei, Taiwan

**Keywords:** physical activity, hemorrhagic stroke, risk factor, cohort study, comorbidities

## Abstract

**Background:** Data on the relationship between physical activity (PA) and hemorrhagic stroke (HS) are limited in Asian populations. This population-based longitudinal follow-up study therefore investigates whether PA is associated with a reduced risk of HS in Taiwan.

**Methods:** A total of 58,857 subjects who had participated in the Keelung Community-based Integrated Screening Program between 2005 and 2012 were enrolled. Information about their PA, obtained using questionnaires, was used to categorize them into three groups according to their average weekly time engaged in it: (1) no PA, (2) low PA (<90 min weekly), and (3) high PA (90 min per week or more). Cox proportional hazard regression was used to evaluate the effect of PA on HS. Stratified analysis by sex and comorbidities (diabetes mellitus, hypertension, and hyperlipidemia) were conducted to evaluate their impact on the relationship between PA and HS.

**Results:** Compared to the no-PA group, the adjusted hazard ratio of HS for the low-PA group was 0.74 (95% CI, 0.57–0.96, *p* = 0.0219), and for the high-PA group, 0.72 (95% CI, 0.58–0.90, *p* = 0.004). The stratified analyses showed that, for the non-comorbidity strata, the beneficial effect of PA on reducing HS risk became stronger as PA increased. However, in the diabetes and hypertension strata, high PA did not appear to have any greater protective effect than low PA.

**Conclusions:** Our findings suggested that even <90 min of PA per week might be beneficial to reduce HS risk. Such a low level of PA is likely to be more achievable and easier to maintain for the general population. Additionally, personalized recommendations based on pre-existing comorbidities may help optimize the beneficial effects of PA on HS prevention.

## Introduction

Stroke is a leading cause of death and disability worldwide. Up to 80% of strokes can be prevented through the management of risk factors such as hypertension, diabetes mellitus (DM) (1), smoking, and physical inactivity (2). Physical activity (PA) is considered one of the most important modifiable risk factors for strokes (3), and in recent years, there has been an increased interest in the effects of PA on stroke prevention. PA may improve vascular function and reduce the risk of stroke by preventing the development of risk factors such as DM and hypertension (4, 5). However, while previous studies have demonstrated that PA may prevent strokes, their evidence base mostly comprises ischemic strokes (6), and data linking PA and hemorrhagic stroke (HS) are relatively limited (7). Moreover, most of the prior studies regarding the relationship between PA and HS were conducted in Western countries, and parallel evidence drawn from Asian populations is even more sparse (4). As well as their different genetic backgrounds, such populations have different lifestyles from their Western counterparts, and their incidence of HS is higher (1). One study from Japan showed that moderate levels of PA were associated with a reduction of approximately 30% in the risk of HS (4). However, a Korean study reported that PA had no significant protective effect against HS (8). Given the limited and inconsistent findings on the relationship between PA and HS in Asian populations, we conducted the present population-based, longitudinal follow-up study to investigate whether PA is associated with a reduced risk of HS and the optimal level of PA for HS prevention in Taiwan. The primary outcome of this study was a new diagnosis of HS retrieved from Taiwan's healthcare database.

## Materials and Methods

### Data Source

This study used data from the Keelung Community-based Integrated Screening program (KCIS), which was conducted by health-service centers in Keelung City, Taiwan (9). The implementation of KCIS started in 1999, and it was originally designed for national neoplastic disease screening. Subjects were invited to participate in it by public-health nurses, and data on their demographic characteristics and lifestyle habits (smoking, drinking, PA, diet, etc.) were collected using structured questionnaires.

The present study used KCIS data from 2005–2012, and linked them to Taiwan's national health insurance research database (NHIRD) and mortality registry for the same 8-year period plus the three following years, i.e., 2013–2015. The NHIRD and mortality-registry data were from the Health and Welfare Data Science Center database at National Taiwan University's Health Data Research Center. Taiwan's national health insurance program is single-payer, compulsory social insurance, and NHIRD is a large-scale healthcare database covering more than 98% of Taiwan's population. Therefore, by linking these databases, a large-scale representative sample of screening data and healthcare records can be collected for analysis. The data that support the findings of this study are available from the corresponding author upon reasonable request.

### Ethics Statement

The present study was approved by the National Taiwan University Hospital Research Ethics Committee. Before analyzing the data, all personal ID numbers in the database were encrypted as alphanumeric codes to protect personal information. The health data used in this study could only be accessed in a privacy-protected room within the Health Data Research Center. Because the data were in de-identified form and analyzed anonymously in a privacy-protected environment, the requirement for informed consent was waived.

### Study Subjects and Covariates

We carried out a longitudinal follow-up study to investigate the impact of PA on HS risk. The questionnaire asked each subject *Are you currently in the habit of engaging in PA?* (Answer options: 0. Never 1. Yes); and those who answered yes were also asked *How many times do you engage in PA in a week?* and *How many minutes is your PA each time?* Based on the answers to these two follow-up questions, each subject's weekly PA time was obtained by multiplying his/her self-reported number of weekly PA sessions by the minutes each one lasted.

**Figure 1:**
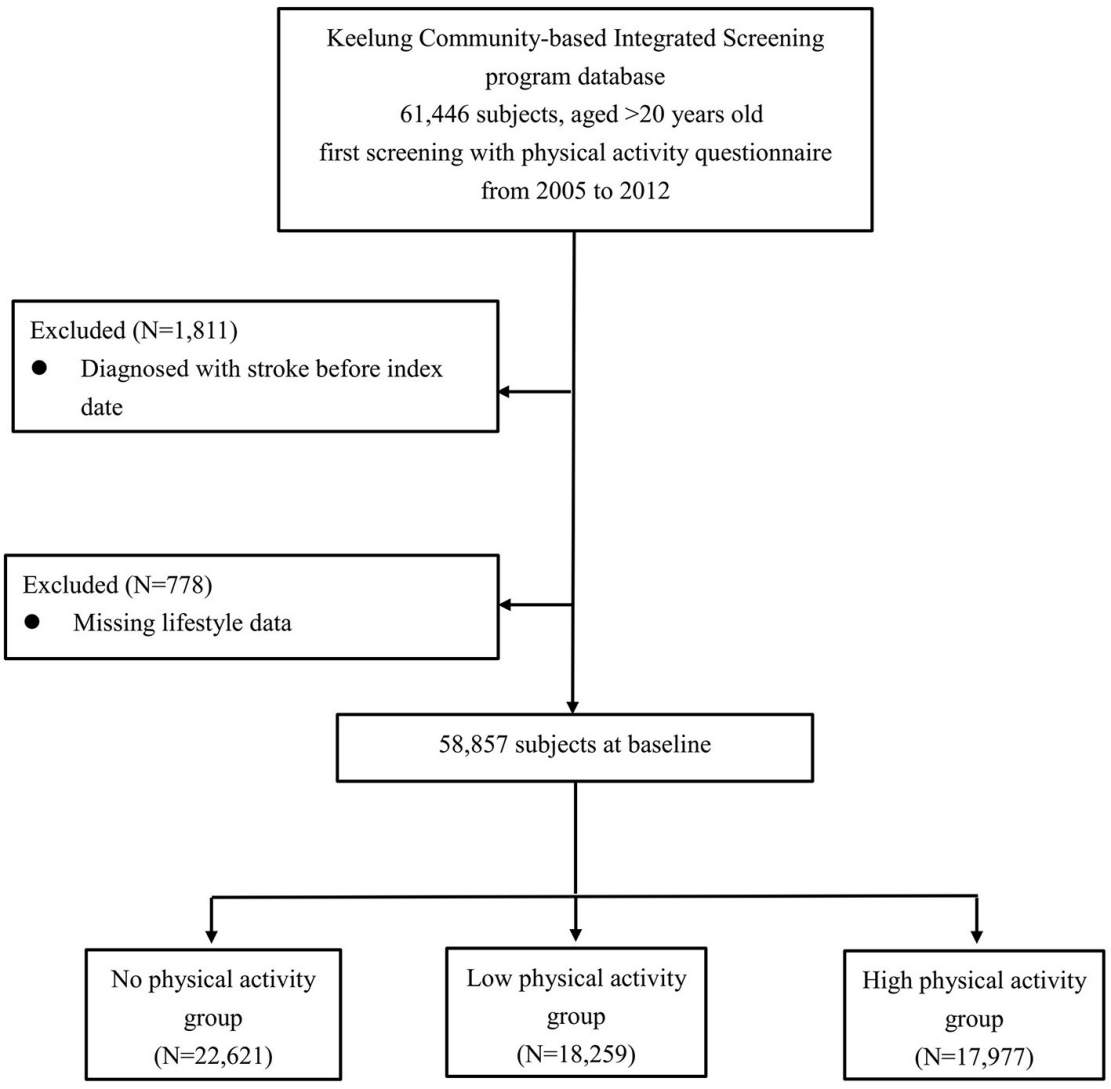
Flow chart illustrating the enrollment process of the study population.

Based on the weekly PA time we calculated, the subjects were categorized into three groups: (1) a no-PA (NPA) group, comprising subjects who answered “Never” to our initial PA question; (2) a low PA (LPA) group, consisting of subjects whose weekly PA times were <90 min; and (3) a high PA (HPA) group, which included subjects whose weekly PA times were 90 min or more. We chose 90 min as the cut-off point between LPA and HPA because a prior population-based study in Taiwan showed that 90 min a week of PA reduced mortality from all causes (10). The recruitment process for the three groups is shown in Figure 1.

We defined the date of initial screening, i.e., questionnaire completion, as the index date. Subjects aged 20 or above at their index dates, provided that those index dates fell within 2005–12, were included (*N* = 61,466). We excluded subjects who had ever been diagnosed with a stroke [International Classification of Diseases, Ninth Revision, Clinical Modification (ICD-9-CM), code 430-438] prior to their respective index dates. After excluding 1,811 subjects who had a history of stroke, 59,655 subjects were left.

As the risk of HS may be affected by demographic factors, lifestyle, and comorbidities (8, 11, 12), these covariates were included in our analysis. The baseline demographic and lifestyle factors collected upon initial screening included sex, age, body mass index, smoking, drinking, and education level. Healthcare records in the NHIRD dating from prior to each patient's the index visit were used to gather information on his/her comorbidities, including DM (ICD-9-CM code 250), hypertension (ICD-9-CM codes 401-405), hyperlipidemia (ICD-9- CM code 272), coronary heart disease (ICD-9-CM codes 410-414, 429.2), atrial fibrillation (ICD-9-CM code 427.31), cancer (ICD-9-CM code 140-208), chronic obstructive pulmonary disease (ICD-9-CM codes 491, 492, 496), osteoarthritis (ICD-9-CM code 715), and chronic kidney disease (ICD-9-CM code 585). To maximize the case ascertainment of such diagnoses, we defined a comorbidity as present if there was at least one discharge record or two outpatient visits with the relevant diagnosis code(s). This case definition has been adopted in previous studies that relied on administrative data (13). Due to missing lifestyle data, a further 778 subjects were excluded, leaving a final sample of 58,857 subjects, who were then divided into the NPA, LPA, and HPA groups as described above.

### Outcome

The primary outcome of this study was a new diagnosis of HS. All outpatient and inpatient healthcare records for each subject were retrieved from the NHIRD. The subjects were tracked from their respective index dates to the earliest of (1) their first occurrence of HS, (2) their death, or (3) the end of 2015. A diagnosis of HS was determined by at least one inpatient discharge or two outpatient visits with a principal diagnostic code of HS (ICD-9-CM codes 430–432). Death dates and causes of death were obtained from the mortality registry.

### Statistical Analysis

We examined the differences in demographic, lifestyle, and comorbidity variables across the three PA groups using chi-square tests and analysis of variance. The incidence rate was calculated as the number of HS events in the group divided by the total follow-up time of the group (per 1,000 person-years). We used the Cox proportional hazard regression analysis to estimate the effects of different PA levels on HS. Since the association between PA and HS may vary with sex and comorbidities, we conducted stratified analysis by sex and comorbidities to evaluate their impact on the association between PA and HS risks (14). An alpha level of 0.05 was considered statistically significant. All analyses were performed using SAS 9.4 software (SAS Institute, Cary, NC).

## Results

Table 1 lists the distribution of baseline demographic factors, lifestyle factors, and comorbidities in the NPA, LPA, and HPA groups. In the LPA group, the median PA time was 45 min per week [interquartile range (IQR) = 30], and in the HPA group it was 210 minutes per week (IQR = 270). There were significant differences in the distribution of baseline characteristics across these three groups (*p* < 0.0001). The average age of the HPA group (52.8 years) was higher than the NPA (44.0 years) and LPA (42.3 years) groups. Moreover, the prevalence of pre-existing comorbidities, such as diabetes mellitus, hypertension, hyperlipidemia, coronary heart disease, cancer, chronic obstructive pulmonary disease, and osteoarthritis, were higher in the HPA group than in the NPA and LPA groups.

**Table 1:** Baseline characteristics of the no physical activity (NPA), low physical activity group (LPA), and high physical activity (HPA) groups.

**Variable**	**NPA (*N* = 22,621)**	**LPA (*N* = 18,259)**	**HPA (*N* = 17,977)**	*p*-value^*^
Sex (women)	14,791 (65.4)	10,790 (59.1)	9,413 (52.4)	<0.0001
Age (years)	44.0 ± 14.4	42.3 ± 13.4	52.8 ± 15.6	<0.0001
Body mass index (kg/m^2^)	24.0 ± 4.2	23.9 ± 3.9	24.5 ± 3.7	<0.0001
Smoking status				<0.0001
Non-smoker	15,754 (69.6)	13,368 (73.2)	13,377 (74.4)	
Ex smoker	1,150 (5.1)	1,284 (7.0)	1,446 (8.1)	
Current smoker	5,717 (25.3)	3,607 (19.8)	3,154 (17.5)	
Drinking				0.0001
Non-drinker	17,480 (77.3)	13,751 (75.3)	13,775 (76.6)	
Ex drinker	746 (3.3)	682 (3.7)	622 (3.5)	
Current drinker	4,395 (19.4)	3,826 (21.0)	3,580 (19.9)	
Education				<0.0001
Less than high school	5,442 (24.1)	2,369 (13.0)	5,676 (31.6)	
High school	10,730 (47.4)	8,185 (44.8)	7,298 (40.6)	
College diploma	6,449 (28.5)	7,705 (42.2)	5,003 (27.8)	
Diabetes mellitus	1,176 (5.2)	766 (4.2)	1,626 (9.0)	<0.0001
Hypertension	2,842 (12.6)	1,914 (10.5)	3,992 (22.2)	<0.0001
Hyperlipidemia	1,627 (7.2)	1,251 (6.9)	2,274 (12.7)	<0.0001
Coronary heart disease	874 (3.9)	514 (2.8)	1,336 (7.4)	<0.0001
Atrial fibrillation	55 (0.2)	42 (0.2)	81 (0.5)	<0.0001
Cancer	262 (1.2)	196 (1.1)	410 (2.3)	<0.0001
COPD	482 (2.1)	292 (1.6)	696 (3.9)	<0.0001
Osteoarthritis	1,282 (5.7)	820 (4.5)	1,819 (10.1)	<0.0001
Chronic Kidney Disease	80 (0.4)	46 (0.3)	95 (0.5)	<0.0001

*Data are presented as N (%) or mean ± standard deviation*.

*COPD, Chronic obstructive pulmonary disease*.

* *Chi-square tests and analysis of variance*.

The three groups' respective numbers of HS events and adjusted hazard ratios (HR) are shown in Table 2. The NPA group collectively experienced 171 HS events (0.98 per 1,000 person-years), the LPA group, 85 (0.60 per 1,000 person-years), and the HPA group, 168 (1.19 per 1,000 person-years). As compared with its NPA counterpart, the LPA group's adjusted HR for the risk of HS was 0.74 (95% CI, 0.57–0.96, *p* = 0.0219), and the HPA group's, 0.72 (95% CI, 0.58–0.90, *p* = 0.004).

**Table 2:** Number of hemorrhagic stroke events and adjusted hazard ratios for the no physical activity (NPA), low physical activity (LPA), and high physical activity group (HPA) groups.

**Variable**	**NPA (*N* = 22,621)**	**LPA (*N* = 18,259)**	**HPA (*N* = 17,977)**
**Hemorrhagic stroke events**, ***N***			
Yes	171	85	168
No	22,450	18,174	17,809
Risk per 1,000 person-year (95% CI)	0.98 (0.84–1.14)	0.60 (0.48–0.75)	1.19 (1.02–1.39)
Adjusted^†^ Hazard ratio (95% CI)	1.00	0.74 (0.57–0.96)^*^	0.72 (0.58–0.90)^*^

*CI, confidence interval*.

* *Cox proportional hazard regression analysis, significant at P <0.05*.

† *Adjusted for sex, age, body mass index, smoking status, drinking, education, diabetes mellitus, hypertension, hyperlipidemia, coronary heart disease, atrial fibrillation, cancer, chronic obstructive pulmonary disease, and chronic kidney disease*.

**Figure 2:**
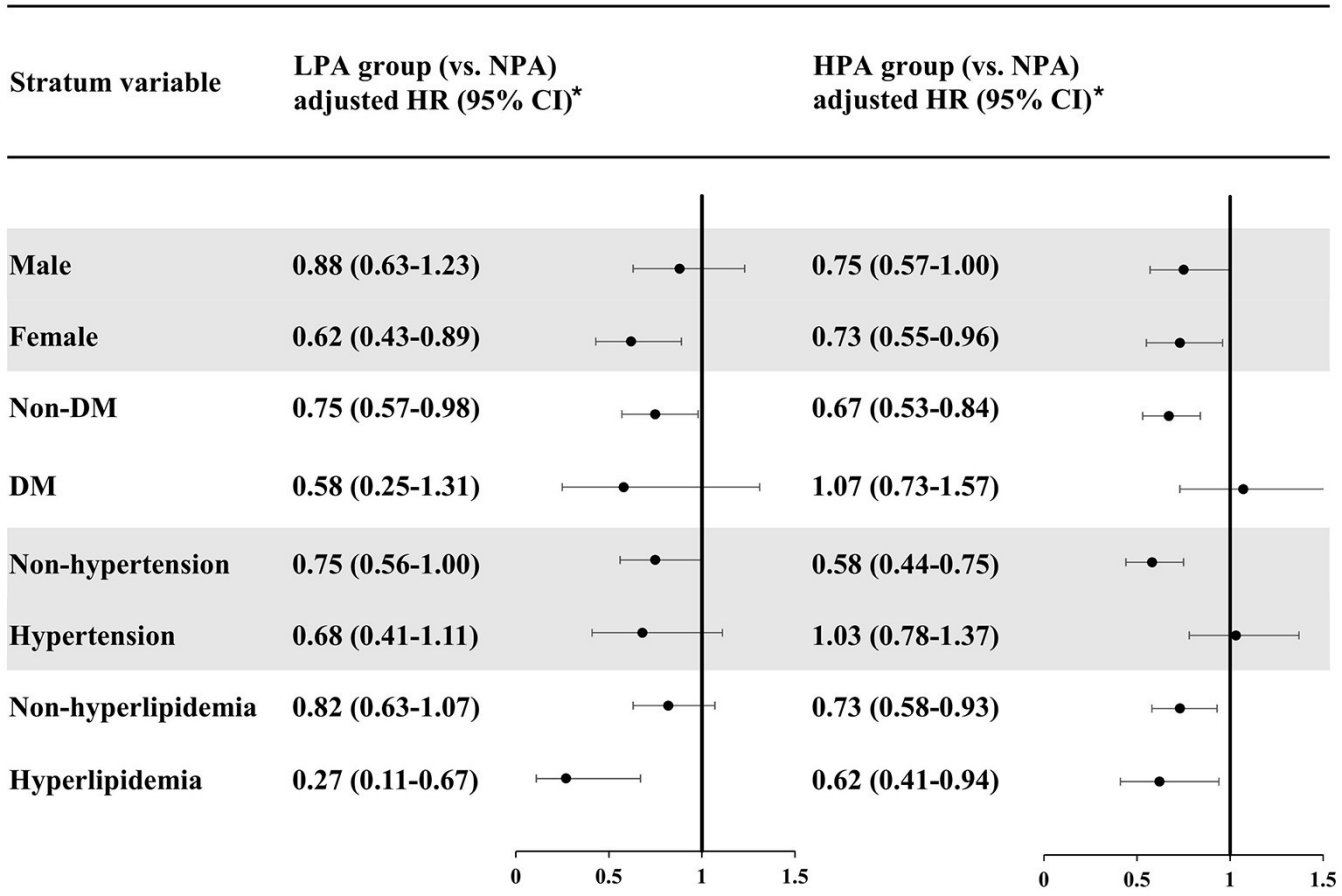
Risk analyses of hemorrhagic stroke, stratified by sex, hypertension, diabetes mellitus, and hyperlipidemia. *Each cox regression model was adjusted for the baseline characteristics in Table 1 except the stratum variable itself. NPA, no physical activity; LPA, low physical activity; HR, hazard ratio; CI, confidence interval; DM, diabetes mellitus.

Figure 2 presents our analysis stratified by sex and comorbidities. With regard to the former, in the women stratum, LPA and HPA had a protective effect against HS (LPA: adjusted HR = 0.62, 95% CI, 0.43–0.89; HPA: adjusted HR = 0.73, 95% CI, 0.55–0.96). In the men stratum, only HPA had a (slight) protective effect on HS (LPA: adjusted HR = 0.88, 95% CI, 0.63–1.23; HPA: adjusted HR = 0.75, 95% CI, 0.57–1.00).

In our analysis stratified by DM (Figure 2), both LPA and HPA had protective effects against HS for the non-DM stratum (LPA: adjusted HR = 0.75, 95% CI, 0.57–0.98; HPA: adjusted HR = 0.67, 95% CI, 0.53–0.84). In the DM stratum, LPA seemed to be associated with a lower risk of HS although lacking statistical significance (adjusted HR = 0.58, 95% CI, 0.25–1.31). The effect of HPA on HS risk was close to null (adjusted HR = 1.07, 95% CI, 0.73–1.57).

In our analysis stratified by hypertension (Figure 2), for the non-hypertension stratum, LPA had a slight protective effect against HS (adjusted HR = 0.75, 95% CI, 0.56–1.00), whereas HPA had a significant protective effect (adjusted HR = 0.58, 95% CI, 0.44–0.75). However, for the hypertension stratum, only the LPA group tended to have a lower risk of HS (adjusted HR = 0.68, 95% CI, 0.41–1.11), and HPA was not associated with a reduced risk of HS (adjusted HR = 1.03, 95% CI, 0.78–1.37).

For the non-hyperlipidemia stratum in our analysis stratified by hyperlipidemia, the LPA group had a slightly lower risk of HS but lacking statistical significance (adjusted HR = 0.82, 95% CI, 0.63–1.07), and being a member of the HPA group was associated with a lower risk of HS (adjusted HR = 0.73, 95% CI, 0.58–0.93). In the hyperlipidemia stratum, on the other hand, both the LPA and HPA groups had a significantly reduced risk of HS (LPA: adjusted HR = 0.27, 95% CI, 0.11–0.67; HPA: adjusted HR = 0.62, 95% CI, 0.41–0.94).

In sum, for the non-DM, non-hypertension, and non-hyperlipidemia strata, the protective effect of PA against HS became stronger as PA time increased. However, in the DM and hypertension strata, HPA was not associated with a lower risk of HS than LPA was.

## Discussion

Although PA has been associated with a reduced risk of stroke (6, 15), the published evidence regarding the association between PA and HS in Asian populations has been limited and inconsistent (4, 8). Moreover, little is known about whether there is a dose-response relationship between PA and HS risk. The present study has shown that LPA and HPA were both associated a reduced risk of HS, as compared with NPA; and, as can be seen in Table 2, this risk reduction appeared to be of a similar magnitude for both LPA and HPA (adjusted HRs, 0.74 and 0.72, respectively). This latter finding that an increase in PA volume does not necessarily lead to a proportional reduction in HS risk seems to be compatible with previous researchers' suggestion that there is a non-linear dose-response relationship between PA and HS risk (4).

For primary prevention of cardiovascular diseases, the World Health Organization recommends ≥150 min per week of moderate intensity or 75 min per week of vigorous-intensity of aerobic PA, or an equivalent mixture of the two (16). The American Heart Association/American Stroke Association guidelines for primary prevention of stroke recommend that healthy adults perform at least moderate-to-vigorous intensity of aerobic PA at least 40 min a day, three to 4 days a week, i.e., 120–160 min per week (17). Our findings, however, imply that <90 min of PA a week might be sufficient to prevent HS, an amount considerably lower than the above-mentioned recommendations, and probably a more achievable goal for most adults. People should therefore be encouraged to take up any level of PA, however low, as a means of gaining the health benefits discussed above.

To our knowledge, only a handful of studies have been published on the relationship between PA and HS risk in Asian populations. One study from Japan reported that moderate levels of PA seemed to be optimal for HS prevention, reducing the risk by ~30% (4). Similarly, in the present study, we found that <90 min a week of PA reduced HS risk by roughly 25% (Table 2). Although detailed information regarding types and intensities of PA was not included in the KCIS data, previous surveys have shown that about half of Taiwan's residents engage in strolling and brisk walking (18, 19). Lower PA times may be perceived as easier goals both to set and to achieve, given that lack of time has been found to be an important barrier to individuals engaging in PA (20).

Likewise, few previous studies have assessed the impact of sex and pre-existing cardiovascular risk factors on the relationship between PA and HS risk. Our stratified analysis by sex (Figure 2) suggests that PA is associated with a lower risk of HS in Taiwanese women, and that the protective effect of PA is stronger for these women than for their male counterparts.

In our analyses that were stratified by DM and by hypertension, PA seemed to have a greater protective effect against HS in the non-DM, non-hypertension strata. In addition, the HPA group experienced greater protective effects than the LPA group in those two strata. However, HPA was no better than LPA when it came to reducing HS risk in the DM and hypertension strata; i.e., there might be no advantage of HPA over LPA in individuals with these two conditions. But the cause of this apparent lack of benefit of more PA is unclear. It has previously been suggested that microangiopathy caused by DM or hypertension can lead to cerebral microbleeds (21), which may increase vulnerability to HS. Moreover, some adverse cardiovascular effects after excessive PA have been sporadically reported (22). Therefore, we hypothesize that DM- or hypertension-related microangiopathy might counteract the potential benefit of HPA in reducing HS risk.

Our analysis stratified by hyperlipidemia, in contrast, showed that both LPA and HPA were associated with more HS risk reduction in the hyperlipidemia stratum than in the non-hyperlipidemia one (Figure 2). Previously, low cholesterol levels have been found to be associated with increased risk of HS (23). Therefore, the protective effect of PA on HS may be counteracted by the potential detrimental impact of low lipid levels on HS risk, and this could explain why the protective effect of PA on HS in the non-hyperlipidemia stratum in our study was less than that in the hyperlipidemia stratum.

According to our analyses stratified by comorbidities (DM, hypertension, hyperlipidemia), the HPA group had a higher magnitude of HS risk reduction than the LPA group in the non-comorbidity strata. Such findings imply that more PA per week should probably be recommended for relatively healthy persons without pre-existing cardiovascular risk factors as a means of HS prevention. In the comorbidity strata, however, the LPA group tended to experience a higher magnitude of HS risk reduction than the HPA group. Therefore, <90 min of PA per week might be a more appropriate approach to HS prevention for persons with pre-existing comorbidities such as hyperlipidemia. Accordingly, personalized recommendations based on the presence or absence of DM, hypertension, and hyperlipidemia may be helpful in optimizing the beneficial impact of PA on HS risk.

Most previous studies conducted in the western countries have reported that participation in PA decreases with age (24, 25). On the contrary, our study showed that the average age of the HPA group was higher than the NPA and LPA groups. Such finding is compatible with other studies conducted in Asia (26–28), and may be explained by increased exercise participation after retirement (29). In addition, because young or middle-aged people typically need to work long hours and take care of family, they may not have much time to engage in regular PA. Another interesting finding is the higher prevalence of pre-existing comorbidities in the HPA group. This finding may also be explained by the higher age in the HPA group, as the prevalence of comorbidities usually increases with age. Moreover, health-seeking behaviors after a disease diagnosis may also increase motivation to engage in PA (26).

The key strength of the present study is its use of a representative, population-based sample with longitudinal follow-up. This enabled us to examine the long-term relationship between PA and the subsequent risk of HS. In addition, we included a variety of comorbid medical conditions in the adjustment for regression analysis. Nonetheless, several limitations should be acknowledged. First, this study used self-reported PA, and detailed information on exercise types and intensities was not available in the original KCIS questionnaires. Therefore, misclassification errors may be present. Second, diagnoses of HS and comorbidities in this study were determined using ICD codes from the NHIRD. This may raise concerns about diagnostic accuracy. However, as well as seeking maximize case ascertainment as described above, we should point out that the Bureau of NHI has formed various audit committees that randomly sample claim data and review the sampled patients' healthcare records so as to optimize diagnostic accuracy and quality of care (30). Third, residents in Taiwan are of Chinese ethnicity, so it is uncertain whether our findings can be generalized to other ethnic groups.

## Conclusions

Asian populations suffer HS more frequently than other populations. The present study has shown that even <90 min a week of PA might help to reduce HS risk in one such population. For most people, this level of PA is likely to be more achievable than the longer times recommended by the WHO and other organizations. Additionally, individualized recommendations regarding PA based on pre-existing comorbidities may help optimize the beneficial effects of PA on HS prevention. A modest amount of PA may be more favorable to lowering HS risk in patients with cardiovascular comorbidities (e.g., hyperlipidemia) than a greater amount. Conversely, more intense and/or lengthy PA may be more conducive to reducing HS risk in people without such comorbidities. However, future studies are needed to confirm our findings.

## Data Availability Statement

The data analyzed in this study is subject to the following licenses/restrictions: The datasets are managed by the Health and Welfare Data Science Center at National Taiwan University's Health Data Research Center. Requests to access these datasets should be directed to ntuhdrc@ntu.edu.tw.

## Ethics Statement

The studies involving human participants were reviewed and approved by National Taiwan University Hospital Research Ethics Committee. Written informed consent for participation was not required for this study in accordance with the national legislation and the institutional requirements.

## Author Contributions

S-HF, L-SC, K-CY, and S-LP designed the research, wrote the manuscript, and conducted the research. S-HF, K-CY, and S-LP analyzed data. All authors have read and approved the final manuscript.

## Funding

This research was supported by a grant from the Ministry of Science and Technology, Executive Yuan, Republic of China (MOST 107-2314-B-002-051-MY2). However, the funder had no role in the study's design, data collection or analysis; the decision to publish it; or the preparation of the manuscript.

## Conflict of Interest

The authors declare that the research was conducted in the absence of any commercial or financial relationships that could be construed as a potential conflict of interest.

## Publisher's Note

All claims expressed in this article are solely those of the authors and do not necessarily represent those of their affiliated organizations, or those of the publisher, the editors and the reviewers. Any product that may be evaluated in this article, or claim that may be made by its manufacturer, is not guaranteed or endorsed by the publisher.
